# Development of a Modern Standard Arabic version of the pain disability index: translation, cross-cultural adaptation, psychometric, and validity data

**DOI:** 10.3389/fmed.2025.1688744

**Published:** 2026-01-08

**Authors:** Leanne Cassidy, Ehab W. Hermena, Eric Francois, Amit Verma, Jaya Batra, Omeesha S. Krishnan, Davide De Marco, Khalifa M. Almenhali, Wadhha J. Alobeidli, Wadhha S. Almuntheri, Kelly L. Huffman

**Affiliations:** 1 Institute for Healthier Living, Abu Dhabi, United Arab Emirates; 2 Department of Psychology, American University of Sharjah, Sharjah, United Arab Emirates; 3 Cleveland Clinic Abu Dhabi, Neurological Institute, Abu Dhabi, United Arab Emirates; 4 Cleveland Clinic Lerner College of Medicine, Cleveland, OH, United States; 5 University of Denver, Denver, CO, United States

**Keywords:** pain disability index, Modern Standard Arabic, chronic pain, psychometric validation, cross-cultural adaptation and validation, patient reported clinical outcomes, health disparities, United Arab Emirates

## Abstract

The assessment and treatment of chronic pain rely heavily on patient self-report, making linguistically and culturally appropriate tools essential. However, no well-validated Arabic language measures of pain-related disability are widely available. The objective of this study was to create and validate a Modern Standard Arabic (MSA) version of the Pain Disability Index (PDI). This prospective cross-sectional study was conducted in a pain management clinic in a tertiary care center in the United Arab Emirates (UAE). The MSA PDI was developed using a forward–backward translation protocol by a team of native Arabic speakers from diverse backgrounds, reviewed by a professional translation company, and pilot-tested with a small sample of patients. Participants completed the MSA PDI along with measures of depression (PHQ-8), anxiety (GAD-7), and current pain severity. A total of 423 Arabic-speaking adults participated (54.84%) women, mean age of 43.71 ± 13.53, most of whom were UAE nationals (88.41%). The mean PDI score was 31.29 (±17.64), indicating moderate pain-related disability. Over half of the sample met screening thresholds for moderate to severe pain (50.83%), depression (57.21%), or anxiety (38.77%). Factor analysis of the MSA PDI supported a unidimensional structure. The MSA PDI also demonstrated excellent internal consistency (α = .91). Construct validity was supported through a tiered multi-method approach (correlation, regression, and structural equation modeling), which showed moderate positive associations between pain severity, depression, anxiety, and pain-related disability. Overall, the MSA PDI showed strong psychometric properties and provides a reliable, standardized tool for assessing pain-related disability in Arabic-speaking populations.

## Introduction

Chronic pain (CP) significantly affects individuals’ physical, psychological, and social wellbeing, and is a recognized global health concern (1). While data from the Arab world are limited, one large-scale study (*n* = 24,265) from Saudi Arabia found that 46.4% of respondents reported chronic pain (2).

The assessment and treatment of chronic pain rely heavily on patient self-report (3), underscoring the need for linguistically and culturally appropriate tools. In multilingual and multicultural settings, language barriers have been shown to negatively influence pain assessment, treatment outcomes, and patient satisfaction. In one study, native Swedish-speaking patients were asked to complete the McGill pain questionnaire (4) in a second language (Finnish), followed by Swedish (5). There were significant discrepancies between responses on the two versions of the McGill, in particular, the affective dimensions of pain. The severity of the mismatch was linked to the patients’ self-rated Finnish proficiency, with higher levels of discordance found in respondents less confident in their Finnish abilities (5). Similarly, another study found that Finnish-speaking patients not only rated their pain as more severe when treated by physicians with lower Finnish language proficiency (6), but also reported lower satisfaction and treatment adherence (6). In the United States, similar studies have focused on native Spanish speakers. One study of children hospitalized postoperatively showed that children with parents with limited English received fewer pain assessments and were less likely to be given opioids, even with similar pain severity ratings (7). Reducing language barriers has been shown to improve care. For example, Spanish-speaking patients provided with consistent access to professional medical interpreters reported better pain control and greater satisfaction with care (8).

Pain-related functional impairment is recommended by the Initiative on Methods, Measurement, and Pain Assessment in Clinical Trials (IMMPACT) as a domain for both research and clinical practice (3). Both patients (9, 10) and physicians (10) prioritize improvements in function as a treatment goal, with some evidence suggesting patients value improved functioning even more than pain relief (11). While pain severity is a reliable predictor of pain-related disability (12, 13), the relationship is modest (14), suggesting additional contributing factors. Psychological variables, such as depression and anxiety that are frequently comorbid with chronic pain, are associated with greater pain severity, increased disability, and lower quality of life (15). Psychological distress may even act as a mechanism through which pain becomes persistent and disabling. Hall et al. (16) found that depression and stress explained 30% of the link between subacute low back pain and later disability.

To date, no widely validated Arabic-language tools exist for assessing pain-related disability. This gap is notable, as Arabic is spoken by ~332 million people in 24 countries (17). In the United States alone, more than 1.4 million people speak Arabic at home (18). The Pain Disability Index (PDI) holds promise as a potential measure to bridge this gap. The PDI is a reliable and valid self-report tool that assesses the impact of chronic pain on daily functioning and quality of life (19). It is valued in clinical and research settings for its simplicity, ability to cover multiple domains of daily life, and patient-friendly format. The PDI has demonstrated strong internal reliability and validity across various populations. It has also been successfully translated into Japanese (20), French Canadian (21), Dutch (22, 23), Turkish (24), supporting the PDI’s conceptual robustness across cultures and languages and potential for Arabic translation.

### Specific aims and hypotheses

This study had two primary aims: (1) to translate the PDI into Modern Standard Arabic (MSA) and (2) to evaluate the psychometric properties of the Arabic PDI, including internal consistency, factor structure, and construct validity.

Our translation process followed a rigorous cross-cultural adaptation process using established guidelines for translation of self-report measures (25, 26). MSA, the formal version of Arabic used in education, government, and healthcare settings across the Arab world, was selected due to its broad comprehension and utility (27). Unlike regional dialects, MSA is universally understood, making it a suitable choice for cross-national use.

Construct validity was explored by examining the relationships of the MSA PDI with other core outcomes recommended by IMMPACT, namely pain severity and emotional functioning (3). Pain severity was captured using a numeric rating scale, and emotional functioning was determined by using Arabic language measures of depression and anxiety. It was hypothesized that MSA PDI would be a reliable, valid measure, and that consistent with previous research, PDI scores would be at least moderately positively associated [*r* ≥ .30, per Cohen’s conventions (28)] with pain severity, depression, and anxiety.

## Materials and methods

### Study design and procedure

This was a prospective cross-sectional study conducted in an outpatient pain management clinic in a tertiary care facility in the United Arab Emirates (Cleveland Clinic Abu Dhabi) between February 2023 and June 2024. While English is the operational language of the hospital, the majority of patients are native Arabic speakers with varying degrees of English proficiency. Non-Arabic-speaking clinicians are supported on-site by professional medical translators.

Potentially eligible patients were identified via review of the electronic medical record (EMR) and were then approached by study staff before or after routine clinic visits. Participants completed informed consent procedures and then were asked to complete three paper-and-pencil measures in Arabic, including the MSA PDI, depression, and anxiety. Measurement of current pain severity was extracted from the EMR as it is a routine standard of care for all clinic visits. Demographic information was extracted either verbally from the patient or from the electronic health record.

#### Ethics

This study was approved by the Cleveland Clinic Abu Dhabi IRB. All participants provided written informed consent.

### Participants

Patients were eligible to participate if they (a) were native Arabic speakers (b) ≥ 18 years of age, and (c) had chronic non-cancer pain lasting longer than 3 months. Patients who were not literate or not able to give informed consent were not eligible for participation in the study; our screening did not identify and exclude any such patients.

Study staff identified 455 potential participants; 26 did not provide informed consent, and six of those identified were determined not to meet the inclusion criteria (cancer pain or acute pain). A total of 423 were enrolled in the study. Participants were 54.84% female (*n* = 232), 62.64% (*n* = 265) married, with a mean age of 43.71(SD = 13.53). The majority (88.41%, *n* = 374) were nationals of the UAE. Tables 1, 2 provide detailed demographics.

**Table 1:** Participant demographics.

Demographics	
Age	
Mean (SD) = 43.71 (13.53)	Range = 18, 84
Gender %, (n)	
Female	54.85 (232)
Male	45.15 (191)
Marital Status %, (n)	
Married	62.65 (265)
Single	30.02 (127)
Divorced	4.49 (19)
Unknown	1.65 (7)
Widowed	0.95 (4)
Separated	0.24 (1)

**Table 2:** Participant nationalities.

Region	Number of participants
GCC countries	380
United Arab Emirates	374
Kuwait & Oman	2 ea
Kingdom of Saudi Arabia & Qatar	1 ea
Other MENA	39
Jordan	11
Egypt	7
Palestine, Syria, & Yemen	4 ea
Iran, Lebanon, & Morocco	2 ea
Iraq, Somalia & Sudan	1 ea
Outside MENA	4
United Kingdom	2
Canada & Pakistan	1 ea

GCC, Gulf Corporation Council; MENA, Middle East North Africa; ea, each.

### Measures

#### Pain-related functional impairment

The PDI is a self-report measure assessing pain’s interference with daily functioning across seven different domains: family responsibilities, recreation, social activity, occupation, sexual behavior, self-care, and life-support activities. Each item is rated on an 11-point scale of 0 (“no disability”) to 10 (“total disability”), with anchors at 3 (“mild disability”), 5 (“moderate disability”), and 8 (“severe disability”). Total scores range from 0 to 70; higher scores indicate greater impairment (19, 29). As there are no empirically driven established cut points for the PDI, we used a straightforward rubric commonly used by clinicians: 0–10 (minimal), 11–20 (mild), 21–30 (mild–moderate), 31–40 (moderate), 41–50 (moderate–severe), 51–60 (severe), and 61 + profound. The PDI is available in the public domain. The PDI has been demonstrated to be both a reliable and valid measure (19, 29, 30). Further, it has been successfully used to evaluate pain-related functional impairment across a wide range of painful conditions, such as acute low back pain (13), and chronic back pain (13, 31, 32), musculoskeletal pain disorders (22), widespread pain (13), breast cancer patients (23), and patients with painful rheumatological disease (24).

#### Translation protocol

Permission to translate the PDI (19) was sought and obtained from the original authors (personal correspondence). The PDI was translated using a forward and backward translation protocol (25, 26), consistent with the COSMIN (consensus-based standards for the selection of health measurement instruments) guidelines (33). Our translation team consisted of native Arabic speakers, with origins in Egypt, Syria, and the United Arab Emirates (UAE), including a physician, a professor, and administrative professionals. The team was selected to ensure linguistic and cultural diversity and to ensure the applicability of the measure across Arabic-speaking populations. The professor, who oversaw the team, has specialized expertise in Arabic psycholinguistics and professional experience in the translation of educational, medical, and therapeutic materials.

First, two independent translators translated the original PDI into Arabic. Discrepancies were reconciled to form a single forward translation. This version was then back-translated into English by two additional translators. The translation team reviewed and reconciled any inconsistencies. As a final step, the resulting Arabic translation was reviewed by an institution-approved professional translation company, in accordance with hospital policy. Minor grammatical revisions were proposed and were acceptable to the team. The edits simplified the reading level of the measure without altering its conceptual content. These edits are detailed in Appendix 1.

The measure was pilot-tested with a small sample (*n* = 30) of patients, following COSMIN recommendations to evaluate the resulting measure from the perspective of the target population (33). No concerns were raised, and no additional changes were made. Appendix 2 contains the resulting Modern Standard Arabic Pain Disability Index.

#### Pain severity

Participants verbally rated current pain intensity on an 11-point numeric rating scale ranging from 0 (“no pain”) to 10 (“worst imaginable pain”) (34). Clinical cutoffs of ≤5, 6–7, and 8 + were used to represent mild, moderate, and severe pain (35).

#### Depression

Depression was measured using a publicly available eight-item Arabic version of the nine-item Patient Health Questionnaire (PHQ-8) (36–38), developed by Pfizer.^1^ The PHQ-9 is a widely used self-report tool based on the DSM-IV criteria (39) for major depression (36–38). The sensitivity and specificity of the PHQ-8 are comparable to the original PHQ-9 (36, 40). Respondents are asked to rate the frequency of depressive symptoms over the past 2 weeks on a scale of 0 *“not at all”* to 3 *“every day.”* Cutoffs of 5, 10, 15, and 20 correspond to mild, moderate, moderately-severe, and severe depression, respectively. A score ≥ 10 is used as a screening threshold for depression (38).

#### Anxiety

Anxiety was measured using the Arabic version of the Generalized Anxiety Disorder scale (GAD-7) (41) developed by Pfizer and publicly available (see text footnote 1). The GAD-7 is based on DSM-IV criteria for generalized anxiety (39). Respondents are asked to rate the frequency of anxiety symptoms over the past 2 weeks on a scale of 0 *“not at all”* to 3 *“nearly every day.”* Total scores range from 0 to 21, with cut-offs of 5, 10, and 15 corresponding to mild, moderate, and severe anxiety, respectively. A score ≥10 has a sensitivity of 89% and a specificity of 82% for generalized anxiety disorder (41).

## Results

### Data analysis

Analyses were conducted in R (version 4.5.0) (42). Full code and output (Appendices 2–6) are available on the OSF platform.^2^

### Factor structure

Assessment of structural validity was conducted using both exploratory and confirmatory factor analysis (EFA and CFA), in line with COSMIN guidelines (33). Due to sample size, EFA and CFA were both conducted using the entire dataset rather than using a split sample approach. The loss in statistical power outweighed the methodological benefit of the split-sample approach for several reasons. First, utilizing the entire dataset approach for both EFA and CFA allowed for ample power to detect misspecification and provide stable parameter estimates. This is essential given that MLR estimation requires larger samples for robust standard errors. Second, the use of FIML to handle missing data further reduces the effective sample size. Finally, we also conducted a split-sample analysis, which confirmed the 1-factor structure reported below. The original unsplit- and the split-sample analyses code and output are available, as in appendices on the OSF platform referenced above.

The suitability of the dataset for factor analysis was assessed using Bartlett’s Test of Sphericity and the Kaiser–Meyer–Olkin (KMO) Measure of Sampling Adequacy (43, 44). Both EFA and CFA were conducted using maximum likelihood (ML) extraction. Missing data were handled using full information maximum likelihood (FIML) estimation, which allows all available data to contribute to parameter estimation without imputation. Missing data were estimated using a saturated model fitted using the Lavaan package in R (45). A correlation matrix based on FIML was extracted and examined.

For EFA, model suitability was examined using the sum of squared loadings (SS loadings), which indicate the proportion of variance explained by each factor. EFA and CFA model fit was evaluated using chi-square (χ^2^), the Tucker–Lewis Index (TLI), root mean square error of approximation (RMSEA), and standardized root mean square residual (SRMR). Fit was interpreted using standard guidelines: RMSEA < 0.05 indicates close fit, 0.05–0.08 acceptable fit, 0.08–0.10 mediocre fit, and > 0.10 poor fit; TLI ≥ 0.95 indicates strong fit; SRMR < 0.08 indicates good fit; and χ^2^/df < 3 is acceptable (46, 47).

EFA was conducted for both one-factor and two-factor models, as prior research supports both structures (19, 30). As the two-factor solution showed poor fit, CFA was conducted with a one-factor model.

### Reliability

Internal consistency reliability was assessed using Cronbach’s alpha and composite reliability (CR), post confirmation of factor structure (33). Cronbach’s alpha was used to evaluate internal consistency, with values ≥ 0.70 considered acceptable (33). CR was computed using standardized factor loadings from the confirmatory factor analysis (CFA) in order to provide a more precise reliability estimate accounting for item-level contributions.

### Construct validity

Construct validity was evaluated by examining the relationships between pain disability and three theoretically related constructs (pain severity, depression, and anxiety) using a multi-method, three-step analytic approach. Our *a priori* hypotheses regarding these relationships [as per COSMIN guidelines (33)] were that pain-related disability would be at least moderately positively associated (*r* ≥ .30) with pain severity, depression, and anxiety.

Convergent validity. To test these hypotheses, we first assessed convergent validity by computing bivariate correlations between total MSA PDI scores and all three constructs. Next, three simple linear regression models were used to assess the predictive value of each construct for total PDI scores. Finally, structural equation modeling (SEM) was used to more rigorously assess these relationships while accounting for measurement error. SEM was conducted for depression and anxiety, as both were measured using multi-item scales (PHQ-8 and GAD-7) and therefore could be modeled as latent variables. Pain disability was modeled as a latent construct using all seven items as indicators. Pain severity was examined only using correlation and regression, as it was assessed with a single item.

For the linear regressions, assumptions of linearity, normality, and homoscedasticity were assessed via residual plots. SEM models were estimated using FIML to handle missing data and the robust maximum likelihood estimator (MLR) to account for non-normality. Model fit was evaluated using the same indices and interpretive criteria described in the factor structure analysis: χ2 , TLI, RMSEA, and SRMR. This tiered analytic strategy provided evidence for construct validity across progressively more complex statistical models.

Known groups analyses. Known-groups analyses (Appendix 3, Known Groups Contrasts) were used to examine the extent to which PDI scores corresponded with established clinical cutoffs for pain severity, depression, and anxiety using t-tests. The Shapiro–Wilk test was used to examine the assumption of normality, and Levene’s test was used to examine the assumption of equal variances.

### Descriptive statistics

There were 361 cases (85.34%) with complete data across all seven PDI items. Missing values across items were as follows: 0.95% (items 1, 6) to 10.40% (*n* = 44, item 5). The PDI was scored if 6 of 7 values were present. Participants who did not answer item 5 were more likely to be female than other participants who completed the item (83.36% vs. 51.19%, *p* < .001) and less likely to be partnered (e.g., single, divorced, or widowed; 27.27% versus 66.75%, *p* < .001). There were no significant differences in median age or mean depression, anxiety, or pain disability index scores.

Scores reflected a wide range of pain-related disability, pain severity, and psychological distress. Mean total PDI score was 31.29 (± 17.64, *n* = 422), consistent with moderate pain-related disability. Mean pain severity was 6.29 (± 6.27); 50.83% (*n* = 215) of participants reported moderate–severe pain (scores ≥ 6). Mean PHQ score was 11.21 (± 6.21); 57.21% (*n* = 242) attained scores consistent with major depression. Mean total GAD score was 9.58 (± 6.01); 38.77% (*n* = 164) attained scores predicting generalized anxiety disorder. In total, 63.59% (*n* = 269) of participants scored above screening thresholds (≥ 10) for either construct. Descriptive statistics are available in Table 3.

**Table 3:** Descriptive statistics.

	Mean (SD)	Range	n
Duration of pain (yrs)	4.65 (5.19)	0.25, 40	423
Pain severity	6.29 (2.31)	0, 10	422
Pain related disability	31.29 (17.64)	0, 70	419
Depression	11.21 (6.21)	0, 24	419
Anxiety	9.58 (6.01)	0, 21	420
Pain related disability % (n)	100 (423)		
Minimal (0–10)	14.89 (63)		
Mild (11–20)	14.42 (61)		
Mild-moderate (21–30)	17.73 (75)		
Moderate (31–40)	18.68 (79)		
Moderate-severe (41–50)	17.97 (76)		
Severe (51–60)	12.06 (51)		
Profound (61–70)	3.31 (14)		
Missing	0.95 (4)		
Pain severity % (n)	100 (423)		
No pain (0)	2.84 (12)		
Mild pain (≤5)	27.90 (118)		
Moderate pain (6–7)	38.06 (161)		
Severe pain (8+)	30.97 (131)		
Missing	0.23 (1)		
Depression % (n)	100 (423)		
Minimal (0–4)	15.84 (67)		
Mild (5–9)	26.00 (110)		
Moderate (10–14)	27.19 (115)		
Moderately severe (15–19)	18.20 (77)		
Severe (20+)	11.82 (50)		
Missing	0.95 (4)		
Anxiety % (n)	100 (423)		
Minimal (0–4)	29.08 (123)		
Mild (5–9)	24.82 (105)		
Moderate (10–14)	19.86 (84)		
Severe (15+)	25.53 (108)		
Missing	3 (0.71)		

yrs, years, SD, standard deviation, pain related disability = score on the translated PDI (29), pain severity = verbal rating scale with clinical cutoffs derived from Boonstra et al. (35); depression = PHQ-8 (38); anxiety = GAD-7 score (41).

### Exploratory factor analysis

Bartlett’s test was highly significant (*p* < .001). The overall KMO value was excellent (0.91) (48). Item-level KMO values ranged between 0.89 and 0.95. The FIML estimated correlation matrix showed adequate correlations across items (Table 4). These findings support the suitability of the dataset for factor analysis.

**Table 4:** Correlation matrix for the seven pain disability index items.

PDI item	PDI_1	PDI_2	PDI_3	PDI_4	PDI_5	PDI_6	PDI_7
PDI_1	1.00						
PDI_2	0.75	1.00					
PDI_3	0.65	0.71	1.00				
PDI_4	0.71	0.68	0.66	1.00			
PDI_5	0.61	0.61	0.62	0.63	1.00		
PDI_6	0.60	0.54	0.61	0.54	0.55	1.00	
PDI_7	0.53	0.49	0.53	0.47	0.49	0.58	1.00

EFA showed that all seven PDI items loaded strongly onto a single factor. The proportion of variance explained by this factor was 60%. SS loadings of 4.21 indicated a well-fitting model. Factor loadings ranged from 0.63 to 0.85, suggesting meaningful contributions from each item. χ^2^ was significant (*p* < .001), suggesting an imperfect model fit. χ^2^, however, is sensitive to sample size; as datasets become larger, even modest model misfit can yield significant *p*-values. Thus, additional model fit indices were examined (RMSR = 0.04, TLI = 0.95, RMSEA = 0.097, 90% *CI* [0.075, 0.12]). While TLI and RMSR indicated good fit, the RMSEA of 0.097 (with upper CI bound of 0.12) suggests mediocre fit according to conventional guidelines.

Next, a two-factor EFA was conducted. The second factor explained only 4% of the variance. Factor loadings for the second factor were weak and did not improve model fit (Table 5). SS loadings were small (0.30). This suggested that the second factor does not represent a meaningful construct. Although prior studies have occasionally proposed two-factor structures (19–24, 29), our findings support previous research demonstrating a one-factor solution (19, 20, 22, 23, 29) as the most appropriate representation.

**Table 5:** Exploratory factor analysis: 1 and 2 factor model item loadings.

PDI items	1 factor		2 factor			
	Factor loadings	Factor communality	Factor 1 loadings	Factor 1 communality	Factor 2 loadings	Factor 2 communality
PDI_1	0.85	0.72	0.84	0.72	−0.11	0.72
PDI_2	0.84	0.71	0.84	0.76	−0.22	0.76
PDI_3	0.82	0.66	0.81	0.66		0.66
PDI_4	0.81	0.66	0.80	0.66	−0.13	0.66
PDI_5	0.75	0.56	0.74	0.55		0.55
PDI_6	0.71	0.50	0.75	0.73	0.41	0.73
PDI_7	0.63	0.40	0.65	0.47	0.24	0.47

The communalities for the two-factor model are identical to those of the 1-factor model, given that the second factor’s loadings are either very small or do not exist (e.g., PDI_3), which means that the contribution from Factor 2 to explaining the variance is negligible.

### Confirmatory factor analysis

Based on EFA results, CFA was conducted using a pre-specified single-factor model. The overall fit of the model was statistically significant (*p* < .001), suggesting that the model did not perfectly reproduce the observed covariance. Other indices indicated mixed results. CFI (0.97) and TLI (0.95) suggested a good fit, with robust versions showing slightly higher values (Robust CFI = 0.97, Robust TLI = 0.96). SRMR was 0.03, well below the recommended cutoff of 0.08, indicating excellent residual fit. However, the RMSEA of 0.08 (90% CI [0.06, 0.10]) indicates mediocre fit according to conventional guidelines (46), with the upper confidence interval bound (0.10) approaching the threshold for poor fit. This discrepancy between fit indices may reflect the scale’s brevity (7 items), as RMSEA can be inflated in models with few degrees of freedom (49).

**Figure 1:**
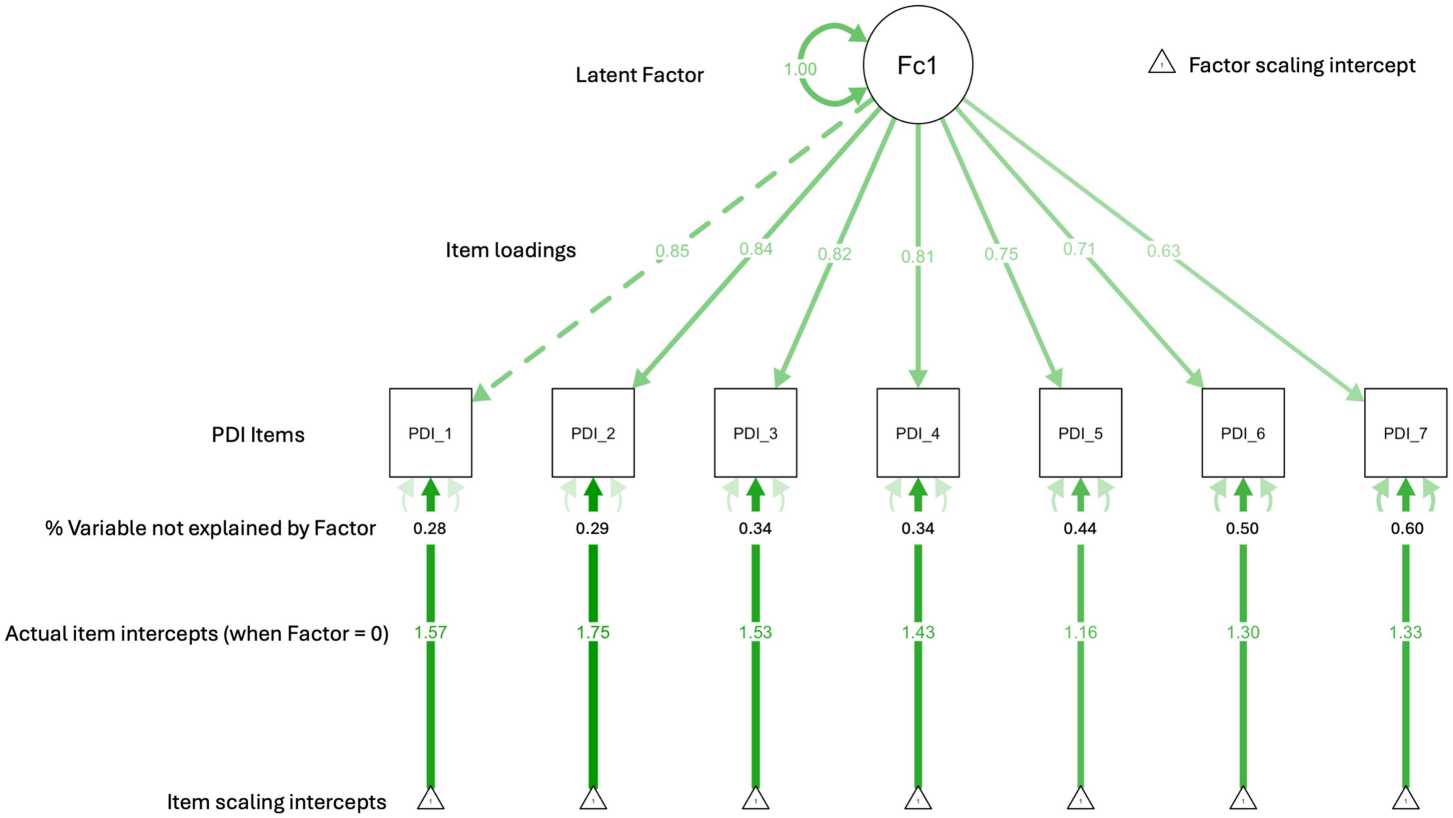
Confirmatory factor analysis: PDI factor structure and item loadings. The dashed line between PDI_1 and the latent factor does not reflect a weaker relationship but rather that PDI_1 is being used as the reference indicator with a fixed loading, and is thus not standardized. The model scaling intercepts (triangles) are all set to the value = I as is customary.

This model explained 60% of the variance in the observed variables. All standardized factor loadings were statistically significant (*p* < .001), ranging from 0.63 (item 7) to 0.85 (item 1), indicating strong relationships between the latent factor and each PDI item (Table 6). Squared multiple correlations (*R^2^*) ranged from .40 to .72, indicating that the single-factor model explains a substantial amount of variance across the PDI items (see Table 6). Figure 1 provides a visual summary of the one-factor structure of PDI, as per the reported CFA. These findings provide further empirical support for the single-factor model as the most robust and parsimonious representation of the data.

**Table 6:** Standardized item loadings (*λ*) and standard error on the factor in the model, along with squared multiple correlations (*R^2^*).

PDI items	*λ*	*SE*	*R^2^*
PDI_1	.85	0.02	.72
PDI_2	.84	0.02	.71
PDI_3	.82	0.03	.67
PDI_4	.81	0.02	.66
PDI_5	.75	0.03	.56
PDI_6	.71	0.03	.50
PDI_7	.63	0.04	.40

### Reliability

Cronbach’s alpha indicated excellent internal consistency (0.91; 95% CI: 0.90–0.92). Removing any individual item, including item 5, sexual behavior, did not affect reliability, as *α* remained above 0.89 in all cases. The average inter-item correlation was 0.60, further supporting internal consistency. Composite reliability (CR) for the single latent factor was 0.91, indicating that one latent construct accounted for a substantial proportion of variance in the observed variables.

### Construct validity

**Figure 2:**
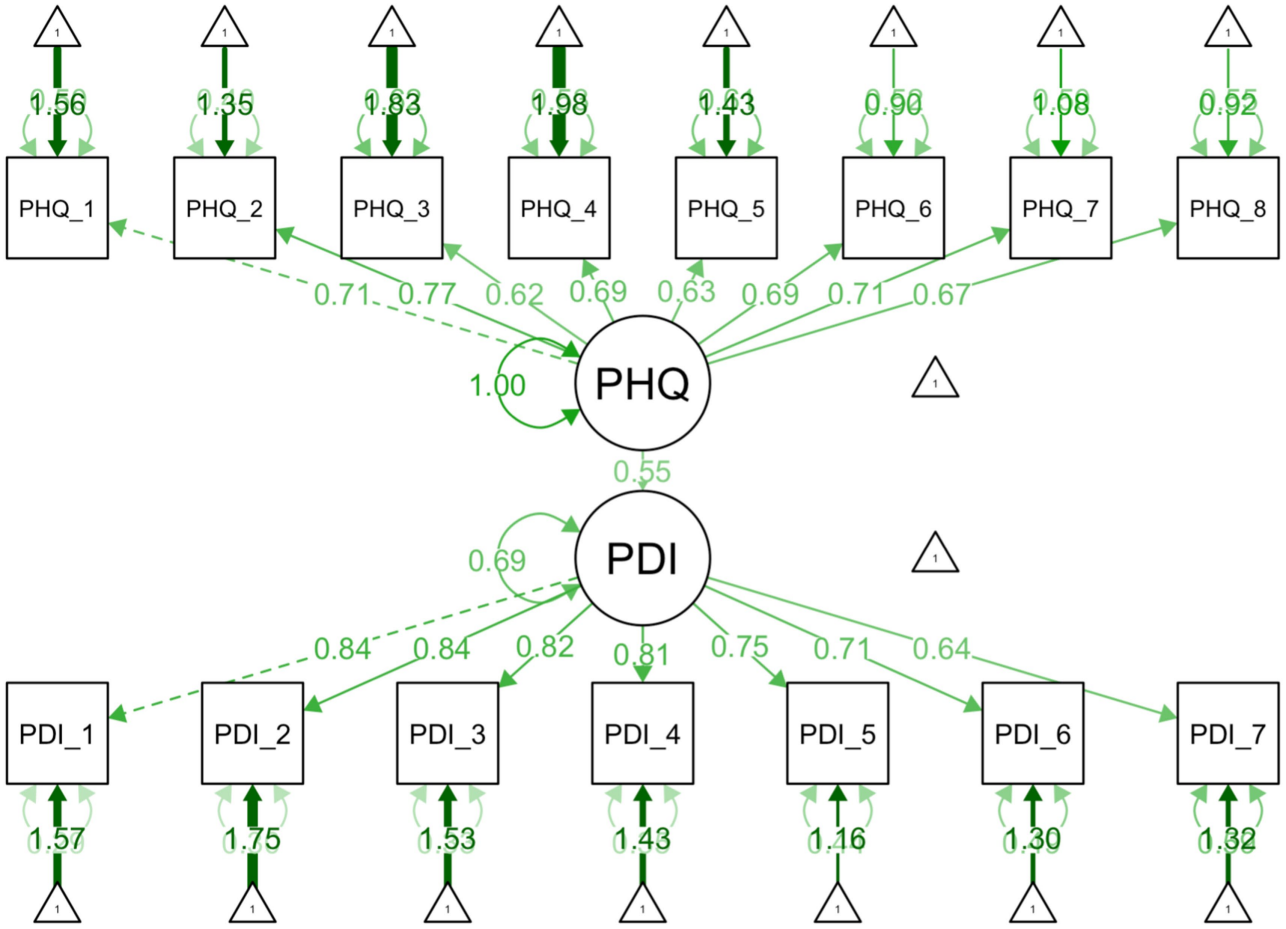
Structural equation model illustrating the relationships between depression and pain related disability. Depression as measured by PHQ-8 and pain related disability as measured by the Arabic PDI.

**Figure 3:**
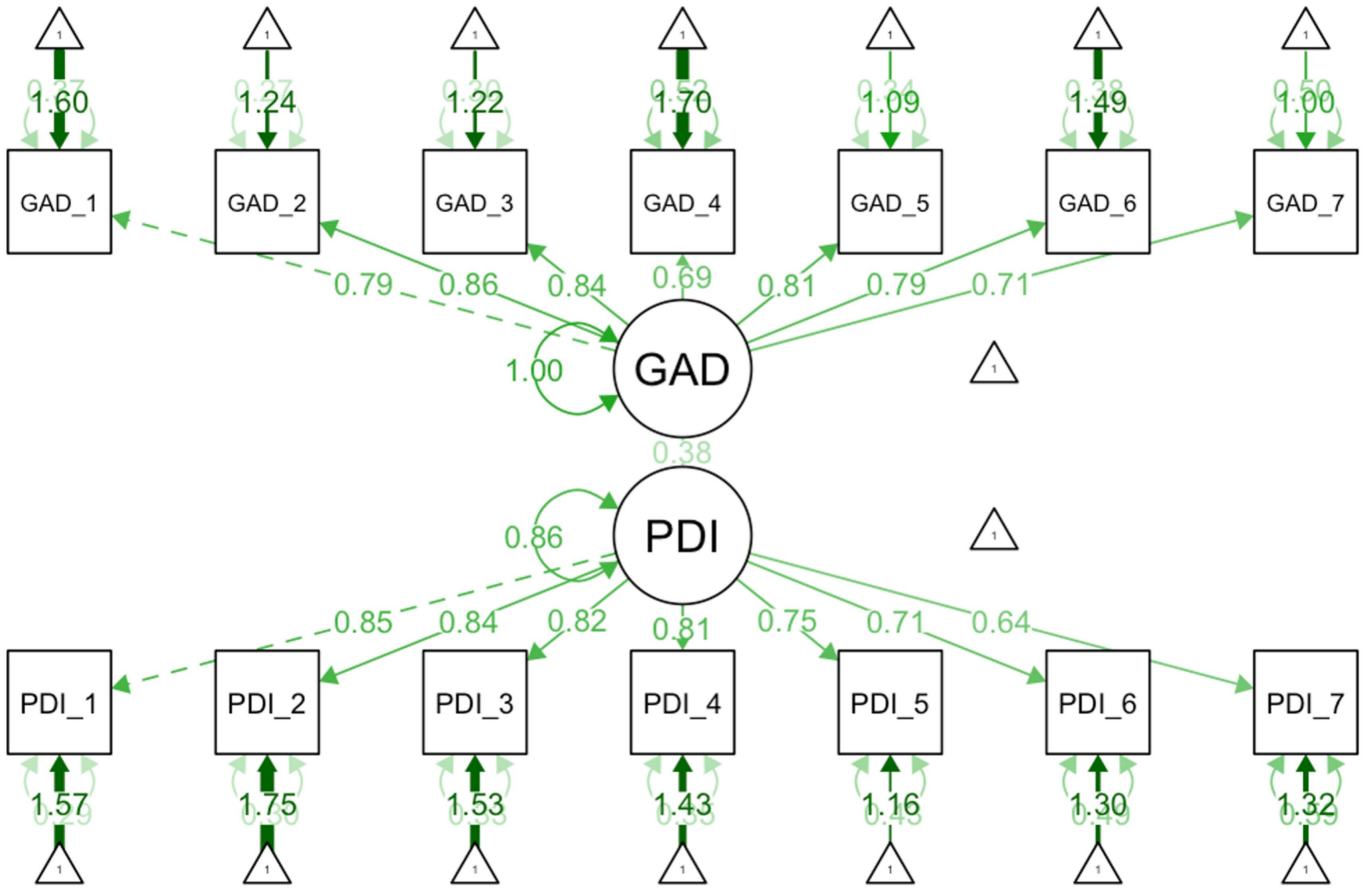
Structural equation model illustrating the relationships between anxiety and pain related disability. Anxiety as measured by GAD-7 and pain related disability as measured by the Arabic PDI.

Assumptions were met (linearity, normality, and homoscedasticity) for all three regressions. For both SEMs, the *χ*^2^ test was significant (*p* < .001), indicating some discrepancies in model fit. This is common in large samples, as discussed, and other model fit indices were acceptable (Appendix 4, EFA-CFA). Detailed regression and SEMS are available in Table 7 and Figures 2, 3.

**Table 7:** Simple linear regressions: pain severity, depression, and anxiety as predictors of pain-related disability.

	B	95% CI	SE	β	*t*	*p*
Pain severity						
Intercept	11.16	6.58, 15.74	2.33	NA	4.79	< .001
Pain severity	3.17	2.49, 3.85	0.35	0.41	9.12	< .001
Depression						
Intercept	15.27	12.24, 18.30	1.54	NA	9.90	< .001
Depression	1.42	1.18, 1.65	0.12	0.50	11.79	< .001
Anxiety						
Intercept	21.52	18.50, 24.53	1.53	NA	14.03	< .001
Anxiety	1.02	0.76, 1.29	0.14	0.35	7.55	< .001

Pain severity: *F*(1, 416) = 83.18, *p* < .001, *R*^2^ = .17, Adj *R*^2^ = .17; Depression: *F*(1, 413) = 139.10, *p* < .001, *R*^2^ = .25, Adj *R*^2^ = .25, Anxiety: *F*(1, 415) = 57.00, *p* < .001; *R*^2^ = .12, Adj *R*^2^ = .12.

Assumption checks for known-groups contrasts demonstrated significant deviations from normality for mild pain severity (VAS ≤ 5) and non-clinical depression/anxiety subgroups (scores >10, *p* < .001). Assumption of equal variance was violated for the anxiety (*p* = .03) and pain severity (*p* = .01) group comparisons only. Given the robustness of Welch’s t-tests to such violations, parametric test statistics were retained in the following analyses.

#### Pain severity

Pain severity was moderately correlated with PDI (*r* = .41). Regression results suggested a significant moderate positive association (*β* = .41), with higher pain intensity predicting higher PDI scores (*p* < .001). Approximately 17% of the variance in PDI was explained by pain severity. Each one-point increase in pain was associated with a 3.17-point increase in PDI score.

Known groups contrasts showed that participants reporting moderate–severe pain levels (VAS > 5) had significantly higher PDI scores (*M* = 35.7, SD = 15.5) than those with mild pain (VAS ≤ 5; M = 21.0, SD = 17.9). This difference was statistically significant, Welch’s *t*(211) = 8.00, *p* < .001. The effect size was large, *d* = 0.90, 95% CI [0.69, 1.12].

#### Depression

Depression was moderately correlated with pain-related disability (*r* = .50) In regression, depression emerged as a strong predictor (*p* < .001), explaining approximately 25% of the variance in PDI, with a moderate effect size (*β* = .50). Each one-point increase in depression was associated with a 1.42-point increase in PDI. In SEM, this relationship remained robust. The structural path from between depression and PDI was significant (*p* < .001), with a moderate–strong effect (*β* = .55). All items significantly loaded on their respective constructs (PHQ = 0.62–0.77; PDI = 0.64–0.84, all *p*s < .001), indicating all items are moderate to strong indicators of their respective latent constructs.

Known group contrasts showed that participants with scores indicative of clinically significant depression (≥10) reported higher pain-related disability (*M* = 37.1, SD = 15.6) than those scoring below the threshold (*M* = 23.0, SD = 16.8). Welch’s *t*-test showed that this difference was statistically significant, Welch’s *t*(352) = −8.64, *p* < .001. The effect size was large, *d* = 0.87, 95% CI [0.67, 1.08] (see Appendix 5).

#### Anxiety

Anxiety was moderately correlated with disability (*r* = .35). Anxiety accounted for 12% of the variance in pain-related disability (*p* < .001). Effect size was moderate (*β* = .35). Each one-point increase in anxiety was associated with a 1.02-point increase in PDI. In the SEM, the standardized path coefficient (*β* = .38) confirmed a moderate effect (*p* < .001). All items loaded strongly on their respective factors (GAD 0.71–0.86; PDI 0.64–0.85, all *p*s < .001), indicating items are moderate to strong indicators of their respective latent constructs.

Known group contrasts showed that participants with scoring above the clinical threshold for anxiety (GAD-7 scores ≥ 10) reported higher PDI scores (*M* = 36.5, SD = 15.9) than those below the threshold (*M* = 26.7, SD = 17.7). Welch’s *t*-test confirmed this difference was statistically significant, Welch’s *t*(409) = −5.95, *p* < .001. The effect size was moderate, *d* = 0.58, 95% CI [0.38, 0.78] (see Appendix 6).

## Discussion

The goal of this study was to translate the PDI into MSA and evaluate its psychometric properties. Pilot testing and patient feedback demonstrated the acceptability and cultural appropriateness of the translated measure. Our data provided strong support for the psychometric properties of the resulting measure, meeting the COSMIN criteria, including structural validity, internal consistency, and construct validity (33).

Psychometric properties were examined using data from 423 individuals. Factor analysis showed all items loaded strongly on a single latent factor, accounting for 60% of the variance. The MSA PDI demonstrated excellent internal consistency and reliability, providing strong evidence that the MSA PDI captures a coherent, single unified construct of pain-related disability. This one-factor model is consistent with two of the three validation studies (19, 29), examining the original English version and prior translations in Dutch and Japanese (20, 22, 23). We did not find support for a two-factor model as found in one previous English language validation study (30) and prior translations in French-Canadian (21) and Turkish (24).

The construct validity of the measure was evaluated using a tiered multi-method analytic approach, including correlational analysis, linear regression, and SEM. All relationships were directionally consistent with expected theoretical relationships, with moderate positive associations between pain disability and related constructs (pain, depression, anxiety), demonstrating that increasing levels of pain, depression, and anxiety are associated with increasing levels of pain-related disability. This is consistent with previous research, which also found significant associations between pain-related functional impairment and both pain severity (20–22, 24, 29, 30) and depression (21, 24, 29–31). In our study, depression was the strongest predictor of pain-related functional impairment, with depression accounting for approximately 25% of the variance in pain-related disability. Pain severity and anxiety also demonstrated significant predictive value, explaining 17 and 12% of the variance, respectively. These findings are in line with established theoretical models of chronic pain that emphasize the interplay of biological, psychological, and social factors.

### Clinical and research implications

The availability of a psychometrically sound MSA PDI holds important implications for improving pain treatment. As language barriers have been shown to influence the accuracy of pain assessment (5) and reduce the quality of care (6), the MSA PDI will be a valuable tool to provide optimal treatment to Arabic-speaking patients, including enhancing diagnostic accuracy, treatment planning, and outcome monitoring. Its excellent reliability suggests it can be used confidently across settings to track treatment response over time. Since MSA is used in healthcare, education, and government across the Arab world, this translation has the potential for cross-national clinical and research applications. The MSA PDI may also facilitate interdisciplinary care by providing a standardized measure that can be used across medical, psychological, and rehabilitative specialties.

Our findings also contribute to the ongoing body of literature examining the interrelationships of psychological distress, pain severity, and pain-related functional impairment. A substantial portion of participants (63.59%) in this study scored above screening thresholds for depression and/or anxiety. This is in line with previous clinical research indicating that persons with chronic pain are more likely to experience depression or anxiety than the general population. The consistent moderately positive associations of these variables with pain-related disability underscore the need for a comprehensive, interdisciplinary approach to pain management. In addition, our work provides additional support for previous research, suggesting that psychological distress may be a more important predictor of disability than pain severity itself. While all three constructs—pain severity, depression, and anxiety—were meaningfully associated with functional impairment, depression was the strongest predictor of pain-related disability. This suggests that psychological interventions play a critical role in improving functional outcomes. Optimal treatment outcomes are likely dependent on addressing co-occurring psychological concerns, whether or not they are a consequence of the pain condition itself.

### Strengths and limitations

A major strength of this study lies in its methodological rigor. The translation process followed established guidelines and included input from linguistically and culturally diverse Arabic-speaking professionals, enhancing its potential generalizability beyond the UAE. Additionally, the use of multiple analytic strategies, including exploratory and confirmatory factor analyses, reliability indices, and structural equation modeling, added depth and robustness to our findings.

Several limitations should be noted. First, EFA and CFA were conducted on the same dataset due to sample size constraints; COSMIN guidelines recommend using independent datasets to minimize bias and confirm structural validity (33). Future studies should aim to replicate these findings in separate samples. Second, it was not feasible to assess test–retest reliability in this study. For pain disability measures, test–retest is typically completed within 7 days, as the score may fluctuate over longer periods due to the natural course of chronic pain and treatment effects. In our setting, participants could only provide data during routine clinical visits, scheduled 1–6 months apart, making it impractical to obtain test–retest data within the recommended timeframe. Future studies are needed to examine the measure’s temporal stability. Third, while most fit indices (CFI, TLI, SRMR) indicated good to excellent model fit, the RMSEA values (0.08–0.097) suggest mediocre fit according to conventional standards (49). This may reflect the scale’s brevity. Future validation studies with larger samples would be of value in further confirming the factor structure of the MSA PDI. Fourth, participants were recruited from a single tertiary care center in the UAE, limiting generalizability. As noted by Turk et al. (14) patients seen in pain clinics differ from those in primary care or the community. It is well documented in the literature that pain clinic populations typically report higher levels of disability, longer pain duration, and greater psychological distress compared to patients in community or primary care samples (50, 51).

Fourth, while the inclusion of depression and anxiety strengthens the evaluation of construct validity, other relevant psychosocial variables [pain catastrophizing (52), self-efficacy (53), and pain coping (54)] were not included. Finally, a significant number of patients did not respond to PDI item 5, which queries sexual behavior. Participants omitting this item were more likely to be women and not formally partnered (e.g., legally married.). The missing data for this item likely reflects cultural and religious beliefs that prohibit any type of sexual behavior outside of marriage. This fact is underscored by the qualitative data provided by participants omitting item 5 (e.g., verbal reports that they did not engage in sexual activity at all, followed by writing NA on the questionnaire itself). Although this has the potential to introduce non-response bias, this pattern has been noted in other studies (22, 55). Importantly, here, inclusion of (or lack thereof) this item did not influence the overall reliability of the measure.

### Future directions

Future studies should replicate these findings in diverse Arabic-speaking populations, including community and primary care samples. Longitudinal research should evaluate the PDI’s sensitivity to clinical change to establish its value as a treatment outcome measure. In addition, future work may explore multivariate models incorporating psychosocial and demographic predictors to clarify the mechanisms linking pain, mood, and disability. Such models could help resolve ongoing debates about the relative contributions of pain intensity and psychological distress and illuminate potential cross-cultural differences in these associations.

## Conclusion

Results demonstrated that the MSA PDI is a reliable and valid measure of pain-related disability, with strong potential for use in clinical and research settings serving Arabic-speaking populations. It addresses a critical gap in pain assessment and supports more inclusive and effective care.

## Data Availability

The datasets presented in this article are not readily available because the data supporting the findings of this study are not publicly available due to institutional and local policy restrictions on patient-level data sharing. De-identified analysis code and output are available via the Open Science Framework repository: https://doi.org/10.17605/OSF.IO/MT9U7. Requests to access the datasets should be directed to kellynnh@gmail.com, Kelly Huffman.

## Footnotes

1
phqscreeners.com

2
https://doi.org/10.17605/OSF.IO/MT9U7

## Supplementary material

All appendices are available on Open Science Framework (OSF): https://doi.org/10.17605/OSF.IO/MT9U7.

## References

[1] Goldberg DS McGee SJ. Pain as a global public health priority. BMC Public Health. (2011) 11:770. doi: 10.1186/1471-2458-11-770, 21978149 PMC3201926

[2] Almalki MT BinBaz SS Alamri SS Alghamdi HH El-Kabbani AO Al Mulhem AA . Prevalence of chronic pain and high-impact chronic pain in Saudi Arabia. Saudi Med J. (2019) 40:1256–66. doi: 10.15537/smj.2019.12.24690, 31828278 PMC6969620

[3] Dworkin RH Turk DC Farrar JT Haythornthwaite JA Jensen MP Katz NP . Core outcome measures for chronic pain clinical trials: IMMPACT recommendations. Pain. (2005) 113:9–19. doi: 10.1016/j.pain.2004.09.012, 15621359

[4] Melzack R. The McGill pain questionnaire: major properties and scoring methods. Pain. (1975) 1:277–99. doi: 10.1016/0304-3959(75)90044-5 1235985

[5] Mustajoki M Forsén T Kauppila T. Pain assessment in native and non-native language: difficulties in reporting the affective dimensions of pain. Scand J Pain. (2018) 18:575–80. doi: 10.1515/sjpain-2018-0043, 29990307

[6] Mustajoki M Forsén T Kauppila T. The association between patient-reported pain and doctors’ language proficiency in clinical practice. Pain Res Treat. (2015) 2015:263904. doi: 10.1155/2015/263904, 26483976 PMC4592902

[7] Jimenez N Jackson DL Zhou C Ayala NC Ebel BE. Postoperative pain management in children, parental english proficiency, and access to interpretation. Hosp Pediatr. (2014) 4:23–30. doi: 10.1542/hpeds.2013-0031, 24435597 PMC4231782

[8] Jimenez N Moreno G Leng M Buchwald D Morales LS. Patient-reported quality of pain treatment and use of interpreters in Spanish-speaking patients hospitalized for obstetric and gynecological care. J Gen Intern Med. (2012) 27:1602–8. doi: 10.1007/s11606-012-2154-x, 22782281 PMC3509300

[9] Wilson L Zheng P Ionova Y Denham A Yoo C Ma Y . CAPER: patient preferences to inform nonsurgical treatment of chronic low back pain: a discrete-choice experiment. Pain Med. (2023) 24:963–73. doi: 10.1093/pm/pnad038, 36975607 PMC12394812

[10] Henry SG Bell RA Fenton JJ Kravitz RL. Goals of chronic pain management: do patients and primary care physicians agree and does it matter? Clin J Pain. (2017) 33:955–61. doi: 10.1097/AJP.0000000000000488, 28244944 PMC5572549

[11] Shanahan ML Rand KL Galloway A Matthias MS. Treatment goals and preferences of black veterans with chronic musculoskeletal pain. J Pain. (2024) 25:104487. doi: 10.1016/j.jpain.2024.02.001, 38336030

[12] Landmark L Sunde HF Fors EA Kennair LEO Sayadian A Backelin C . Associations between pain intensity, psychosocial factors, and pain-related disability in 4285 patients with chronic pain. Sci Rep. (2024) 14:13477. doi: 10.1038/s41598-024-64059-8, 38866885 PMC11169509

[13] Gronblad M Hupli M Wennerstrand P Järvinen E Lukinmaa A Kouri JP . Intercorrelation and test-retest reliability of the pain disability index (PDI) and the Oswestry disability questionnaire (ODQ) and their correlation with pain intensity in low back pain patients. Clin J Pain. (1993) 9:189–95. doi: 10.1097/00002508-199309000-00006, 8219519

[14] Turk DC. Clinical effectiveness and cost-effectiveness of treatments for patients with chronic pain. Clin J Pain. (2002) 18:355–65. doi: 10.1097/00002508-200211000-00003, 12441829

[15] Bair MJ Wu J Damush TM Sutherland JM Kroenke K. Association of depression and anxiety alone and in combination with chronic musculoskeletal pain in primary care patients. Psychosom Med. (2008) 70:890–7. doi: 10.1097/PSY.0b013e318185c510, 18799425 PMC2902727

[16] Hall AM Kamper SJ Maher CG Latimer J Ferreira ML Nicholas MK. Symptoms of depression and stress mediate the effect of pain on disability. Pain. (2011) 152:1044–51. doi: 10.1016/j.pain.2011.01.014, 21306826

[17] Eberhard DM Simons GF Fennig CD (eds). Ethnologue: languages of the world, 27th ed. Dallas, TX: SIL International. (2024). Available online at: http://www.ethnologue.com (Accessed June 1, 2025).

[18] Pew Research Center. (2023). 5 facts about Arabic speakers in the U.S. Available online at: https://www.pewresearch.org/short-reads/2023/05/18/5-facts-about-arabic-speakers-in-the-us/#:~:text=The%20number%20of%20people%20ages,those%20born%20in%20the%20U.S (Accessed June 1, 2025).

[19] Chibnall JT Tait RC. The pain disability index: factor structure and normative data. Arch Phys Med Rehabil. (1994) 75:1082–6. doi: 10.1016/0003-9993(94)90082-5, 7944912

[20] Yamada K Mibu A Kogo S Sullivan M Nishigami T. Reliability and validity of the Japanese version of pain disability index. PLoS One. (2022) 17:e0274445. doi: 10.1371/journal.pone.0274445, 36094940 PMC9467349

[21] Gauthier N Thibault P Andams H Sullivan MJL. Validation of a French-Canadian version of the pain disability index. Pain Res Manag. (2008) 13:327–33. doi: 10.1155/2008/461436 18719715 PMC2671319

[22] Soer R Köke AJA Vroomen PCAJ Stegeman P Smeets RJEM Coppes MH . Extensive validation of the pain disability index in 3 groups of patients with musculoskeletal pain. Spine. (2013) 38:E562–8. doi: 10.1097/BRS.0b013e31828af21f, 23388675

[23] Van der Gucht E Dams L Bernar K De Vrieze T Haenen V De Groef A . The Dutch language version of the pain disability index (PDI-DLV): psychometric properties in breast cancer patients. Physiother Theory Pract. (2023) 39:2000–14. doi: 10.1080/09593985.2022.2059036, 35378054

[24] Uğurlu M Uğurlu GK Erten Ş Ulusoy Kaymak S Çayköylü A. Reliability and factorial validity of the Turkish version of the pain disability index in rheumatic patients with chronic pain. Arch Rheumatol. (2016) 31:265–71. doi: 10.5606/ArchRheumatol.2016.5750, 29900947 PMC5827852

[25] Guillemin F Bombardier C Beaton D. Cross-cultural adaptation of health-related quality of life measures: literature review and proposed guidelines. J Clin Epidemiol. (1993) 46:1417–32. doi: 10.1016/0895-4356(93)90142-N, 8263569

[26] Tsang S Royse CF Terkawi AS. Guidelines for developing, translating, and validating a questionnaire in perioperative and pain medicine. Saudi J Anaesth. (2017) 11:80–S89. doi: 10.4103/sja.SJA_203_17, 28616007 PMC5463570

[27] Versteegh K. The Arabic language. 2nd ed. Edinburgh: Edinburgh University Press (2014).

[28] Cohen J. Statistical power analysis for the behavioural sciences. 2nd ed. Hillsdale, NJ: Lawrence Erlbaum Associate (1988).

[29] Tait RC Chibnall JT Krause S. The pain disability index: psychometric properties. Pain. (1990) 40:171–82. doi: 10.1016/0304-3959(90)90068-O, 2308763

[30] Tait RC Pollard CA Margolis RB. The pain disability index: psychometric and validity data. Arch Phys Med Rehabil. (1987) 68:438–41. 3606368

[31] Soer R Reneman MF Vroomen PCAJ Stegeman P Coppes MH. Responsiveness and minimal clinically important change of the pain disability index in patients with chronic back pain. Spine. (2012) 37:711–5. doi: 10.1097/BRS.0b013e31822c8a7a, 21796022

[32] Soer R Köke AJA Speijer BLGN Köke AJ Speijer BL Vroomen PC . Reference values of the pain disability index in patients with painful musculoskeletal and spinal disorders: a cross-national study. Spine (Phila Pa 1976). (2015) 40:E545–51. doi: 10.1097/BRS.0000000000000827, 26030221

[33] Mokkink LB Terwee CB Patrick DL Alonso J Stratford PW Knol DL . The COSMIN checklist for assessing the methodological quality of studies on measurement properties of health status measurement instruments: an international Delphi study. Qual Life Res. (2010) 19:539–49. doi: 10.1007/s11136-010-9606-8, 20169472 PMC2852520

[34] Hartrick CT Kovan JP Shapiro S. The numeric rating scale for clinical pain measurement: a ratio measure? Pain Pract. (2003) 3:310–6. doi: 10.1111/j.1530-7085.2003.03034.x, 17166126

[35] Boonstra AM Stewart RE Albère AJ Köke AJA Oosterwijk RFA Swaan JL . Cut-off points for mild, moderate, and severe pain on the numeric rating scale for pain in patients with chronic musculoskeletal pain: variability and influence of sex and catastrophizing. Front Psychol. (2016) 7:1466. doi: 10.3389/fpsyg.2016.01466 27746750 PMC5043012

[36] Kroenke K Spitzer RL. The PHQ-9: a new depression diagnostic and severity measure. Psychiatr Ann. (2002) 32:509–15. doi: 10.3928/0048-5713-20020901-06, 37337024

[37] Kroenke K Spitzer RL Williams JBW. The PHQ-9: validity of a brief depression severity measure. J Gen Intern Med. (2001) 16:606–13. doi: 10.1046/j.1525-1497.2001.016009606.x, 11556941 PMC1495268

[38] Kroenke K Strine TW Spitzer RL Williams JBW Berry JT Mokdad AH. The PHQ-8 as a measure of current depression in the general population. J Affect Disord. (2009) 114:163–73. doi: 10.1016/j.jad.2008.06.026, 18752852

[39] American Psychiatric Association. Diagnostic and statistical manual of mental disorders. 4th ed. Washington DC: American Psychiatric Association. (2000).

[40] Corson K Gerrity MS Dobscha SK. Screening for depression and suicidality in a VA primary care setting: 2 items are better than 1 item. Am J Manag Care. (2004) 10:839–45. 15609737

[41] Spitzer RL Kroenke K Williams JBW Löwe B. A brief measure for assessing generalized anxiety disorder: the GAD-7. Arch Intern Med. (2006) 166:1092–7. doi: 10.1001/archinte.166.10.1092, 16717171

[42] R Core Team. A language and environment for statistical computing [computer program]. Version 4.5.0. Vienna Austria: R foundation for statistical computing. (2023). Available online at: https://www.R-project.org/ (Accessed June 1, 2025).

[43] Hair JF Babin BJ Anderson RE Black WC. Multivariate data analysis, 8th ed. Boston, MA: Cengage (2018).

[44] Field AP. Discovering statistics using IBM SPSS Statistics. 5th ed. London: SAGE Publications. (2018).

[45] Rosseel Y. Lavaan: an R package for structural equation modeling. J Stat Softw. (2012) 48:1–36. doi: 10.18637/jss.v048.i02

[46] Hu LT Bentler PM. Cutoff criteria for fit indexes in covariance structure analysis: conventional criteria versus new alternatives. Struct Equ Modeling. (1999) 6:1–55. doi: 10.1080/10705519909540118

[47] Kline RB. Principles and practice of structural equation modeling. 4th ed. London: The Guilford Press (2016).

[48] Kaiser HF. An index of factorial simplicity. Psychometrika. (1974) 39:31–6. doi: 10.1007/BF02291575

[49] Browne MW Cudeck R. Alternative ways of assessing model fit. Sociol Methods Res. (1992) 21:230–58. doi: 10.1177/0049124192021002005

[50] Crook J Weir R Tunks E. An epidemiological follow-up survey of persistent pain sufferers in a group family practice and specialty pain clinic. Pain. (1989) 36:49–61. doi: 10.1016/0304-3959(89)90111-5, 2919095

[51] Crook J Tunks E Rideout E Browne G. Epidemiologic comparison of persistent pain sufferers in a specialty pain clinic and in the community. Arch Phys Med Rehabil. (1986) 67:451–455. 3741544

[52] Severeijns R Vlaeyen JW van den Hout MA Weber WE. Pain catastrophizing predicts pain intensity, disability, and psychological distress independent of the level of physical impairment. Clin J Pain. (2001) 17:165–72. doi: 10.1097/00002508-200106000-00009, 11444718

[53] Turner JA Ersek M Kemp C. Self-efficacy for managing pain is associated with disability, depression, and pain coping among retirement community residents with chronic pain. J Pain. (2005) 6:471–9. doi: 10.1016/j.jpain.2005.02.011, 15993826

[54] Turner JA Clancy S. Strategies for coping with chronic low back pain: relationship to pain and disability. Pain. (1986) 24:355–64. doi: 10.1016/0304-3959(86)90121-1, 2938059

[55] Gross DP Knupp H Esmail S. The utility of measuring sexual disability for predicting 1-year return to work. Arch Phys Med Rehabil. (2011) 92:1870–4. doi: 10.1016/j.apmr.2011.06.020, 22032221

